# Rezension „Wie Corona die Hochschullehre verändert“

**DOI:** 10.1365/s40702-021-00791-3

**Published:** 2021-09-27

**Authors:** Linda Grogorick

**Affiliations:** Institut für Wirtschaftsinformatik, TU Braunschweig, Braunschweig, Deutschland

## *Ullrich Dittler, Christian Kreidl*

**„Wie Corona die** **Hochschullehre verändert“**

ISBN 978-3-658-32608‑1, Springer Gabler, Wiesbaden, 406 S., 54,99 €

„Wer Bücher liest, schaut in die Welt und nicht nur bis zum Zaune“

Getreu dem Zitat, welches dem deutschen Dichter Johann Wolfgang von Goethe zugeordnet wird, ermöglicht der Sammelband „Wie Corona die Hochschullehre verändert“ einen Blick über den Tellerrand, indem verschiedene Perspektiven sowie Herangehensweisen und Erfahrungen des Sommersemester 2020 unterschiedlicher Hochschulen präsentiert werden. Bedingt durch Covid-19 mussten sich Hochschulen im Frühjahr 2020 der Herausforderung stellen zumindest temporär auf Präsenzlehre zu verzichten und ihre gesamten Lehrangebote augenblicklich ins Digitale zu überführen.

Ziel des Sammelbands der herausgebenden Autoren Ullrich Dittler und Christian Kreidl ist es vor diesem Hintergrund verschiedene Perspektiven auf diese besondere Situation zu zeigen und zusammenzuführen, wobei Lehrende an Hochschulen der Länder Deutschland, Österreich und Schweiz Einblicke ermöglichen.

Der Sammelband enthält 24 Beiträge, wobei 44 Autorinnen sowie Autoren mitwirken und unterschiedliche Sichtweisen auf dieses besondere Semester präsentieren. Jeder Beitrag bildet dabei ein eigenständiges Kapitel im Buch, so dass diese sowohl sequenziell, aber auch punktuell gelesen werden können, je nachdem, was in der Situation und dem Moment des Lesens relevant für einen selbst ist. Persönlich hätte ich mir hier eine zusätzliche weitere Aufteilung in thematische Parts gewünscht, so dass verschiedene Beiträge zu einem bestimmten Themenschwerpunkt, wie beispielsweise zur Sichtweise der Lehrenden, zusammengefasst und in einem kurzen Einführungstext die Verbindungen zwischen den verschiedenen enthaltenen Beiträgen aufgezeigt werden. Dies ist aber Geschmackssache. Schließlich ermöglicht die gewählte Form ein losgelöstes Denken von festen Strukturen, wodurch die Entwicklung eigener Assoziationen optimal gefördert wird.

Die präsentierten Perspektiven und Inhalte der 24 Kapitel sind äußerst vielfältig, weshalb nachfolgend ein kurzer Überblick gegeben wird. Den Einstieg in das Buch bildet eine kurze Einführung in die rasante Verbreitung von Covid-19, um einen Rahmen und gleichzeitig ein Bewusstsein dafür zu schaffen, weshalb und wie sich die Lehre an den Hochschulen massiv verändern musste (Kap. 1). Daraufhin widmen sich die folgenden zwei Kapitel der Vorstellung ausgewählter Ergebnisse aus einer quantitativen Studie mit mehr als 3500 teilgenommenen Studierenden (Kap. 2) und einer ergänzenden qualitativen Studie mit zehn durchgeführten Interviews (Kap. 3). Beide Kapitel zusammen liefern den Leserinnen und Lesern einen runden Eindruck von der Wahrnehmung der Studierenden, der größten betroffenen Gruppe der Hochschulangehörigen, während dieser Zeit. Im darauffolgenden Kapitel zeigen zwei Autorinnen die Bedeutung von Vernetzung, Zusammenarbeit und Diskurs für die Gestaltung von Hochschullehre auf (Kap. 4). Der Prozess und die Sichtweise der Hochschulleitung bei der abrupten Überführung von Präsenzlehre zu einem vollständigen digitalen Lehrangebot wird ebenfalls in einem Kapitel erläutert (Kap. 5). Ein weiteres Kapitel basiert auf den Ergebnissen zweier empirischer Untersuchungen, in denen die Veränderungen des Lehrens und Lernens in Folge von Covid-19 aus Studierenden- und Lehrendensicht betrachtet werden (Kap. 6). Ebenfalls sind ergänzend die Perspektiven der praxisorientierteren Hochschulen interessant (Kap. 7). Notwendige Unterstützung bei der plötzlichen Umstellung ins Digitale, die eher im Hintergrund ablief, und beispielsweise zentrale Einrichtungen oder das Rechenzentrum betrifft, thematisiert ein Beitrag der Ludwig-Maximilians-Universität München (Kap. 8). Eine Erklärung der notwendigen Vorbereitungen für ein rein digitales Semester, dessen Planung in wenigen Wochen erfolgen musste, findet in Kap. 9 statt. Ausgehend von den bisherigen Erfahrungen beim digitalen Lernen erhalten die Leserinnen und Leser aus der Sicht eines Prorektors Einblicke, wie diese die Grundlage für das Corona-Semester gebildet haben (Kap. 10). Zunächst werden im Kap. 11 die Potenziale, die schon lange mit digitaler Lehre einhergehen vorgestellt, bevor die Ergebnisse zweier Umfragen präsentiert werden, die beweisen, dass das Online-Semester weitestgehend positiv evaluiert wurde. Die ermöglichten Freiheiten bei der Durchführung vollständiger digitaler Lehre, bei der eine der größten Herausforderungen die Kontaktermöglichung zwischen den Studierenden ist, stehen in Kap. 12 am Beispiel der Hochschule Mannheim im Fokus. Nachfolgend zeigen Interviews mit Expertinnen und Experten aus der Lehre und dem Hochschul-Management aus Deutschland ein Stimmungsbild des Corona-Semesters (Kap. 13). Getreu dem Ausruf „Krise ist auch immer eine Chance!“ betrachtet der Autor des Kap. 14, wie schnell und eindrucksvoll in dem Corona-Semester die Bemühungen des digitalen Lehrens und Lernens geglückt sind, nachdem es bereits seit den 1990er-Jahren entsprechende Bestrebungen mit eher durchwachsenen Erfolg gab. Im darauffolgenden Kapitel werden unterstützende sowie hemmende Faktoren von Digitalisierungsvorhaben identifiziert (Kap. 15). Des Weiteren wird der „ReDesign-Canvas“ als Hilfsmittel für die didaktische und methodische Neugestaltung von Lehrveranstaltungen vorgestellt (Kap. 16). Eine Reflektion des Einsatzes von Videos und PodCasts zu Lehr- und Lernzwecken erfolgt in Kap. 17. Zur Aneignung von Medienkompetenz wurde vor dem Hintergrund von Covid-19 ein besonderes Seminar zur Beobachtung, Analyse und Reflexion der Geschehnisse aus Studierendensicht angeboten, welches näher in Kap. 18 beschrieben ist. Interviews mit Expertinnen und Experten aus der Lehre und dem Hochschul-Management aus Österreich zeigen darüber hinaus ergänzend zu den Interviews in Kap. 13 ebenfalls ein Stimmungsbild des Corona-Semesters (Kap. 19). Mit dem digitalen Semester gingen auch digitale Prüfungen einher, weshalb im Kap. 20 Rahmenbedingungen und Gründe für bzw. gegen die Teilnahme an einer Online-Klausur mithilfe einer Befragung der Studierenden thematisiert werden. Auch die Qualitätssicherung des Studiums und der Lehre im Allgemeinen sind wesentliche Schwerpunkte der Hochschulen. Daher werden im folgenden Kapitel Ergebnisse einer Studierendenbefragung zur wahrgenommenen Qualität der digitalen Lehre vorgestellt (Kap. 21). Einen deskriptiven Vergleich zwischen Deutschland und der Schweiz ermöglichen zwei Befragungen von Lehrenden, in denen die Erfahrungen mit der Umstellung auf digitale Lehre erhoben worden sind (Kap. 22). Vor dem Hintergrund des VUCA-Umfelds, geprägt durch Volatilität, Unsicherheit, Komplexität und Mehrdeutigkeit, ist digitale Lehre bedingt durch Covid-19 praktisch über Nacht zum Standard geworden. Kap. 23 widmet sich daher der Thematik, wie Corona das Lernen und Lehren an Hochschulen verändert hat und insbesondere weiterhin verändern wird, bevor im abschließenden Kap. 24 die innerhalb kürzester Zeit realisierten digitalen Lernangebote reflektiert und vor allem kritisch hinterfragt werden, um zu evaluieren, ob die Lehre im Allgemeinen den Herausforderungen des 21. Jahrhunderts gerecht wird.

Insgesamt gelingt es den beiden Herausgebern des Sammelbands gemeinsam mit den Autorinnen und Autoren der einzelnen Kapitel unterschiedliche Herangehensweisen und Entwicklungen zu dokumentieren, wodurch den Leserinnen und Lesern die Chance eröffnet wird nicht nur die eigene Lehre und der eigene Umgang während dieser Zeit zu reflektieren, sondern auch neue Impulse und Anregungen für die Gestaltung der Lehre und Prozesse an der eigenen Hochschule zu identifizieren. Doch dieser Sammelband liefert nicht nur für den Hochschulkontext spannende Einblicke, sondern auch für die Praxis insbesondere unter dem Aspekt des lebenslangen Lernens, nach dem eine kontinuierliche (digitale) Weiterbildung auch im Berufsalltag relevant und die Grundlage für die Stärkung der Wettbewerbsfähigkeit sowie der Schaffung von Innovationen ist.

